# A Fish-hook Removed from the Œsophagus without an Operation

**Published:** 1845-04

**Authors:** Andrew R. Kilpatrick

**Affiliations:** Woodville, Miss.


*A Fish-hook removed from the Œsophagus without an operation. Reported by* ANDREW R. KILPATRICK, M. D., *Woodville, Miss.*—In the summer of 1837, Mrs. * * * was enjoying her usual *siesta*, in the afternoon of a warm day, on a pallet spread upon the floor in a cool part of the house:—and while she was lying on her back sleeping pleasantly, no doubt dreaming of past pleasures, her grandson, a little urchin of three or four summers, was playing about the house with a fishing tackle complete, pole, line and hook; who, when he discovered the old lady with her mouth widely distended, thought it was a fine opportunity to "catch a fish." Accordingly, in order to effect his purpose, he cautiously deposited the "barbed hook," (I believe there was no *bait* on it,) into his granddame's open mouth. The titilation caused her to awake suddenly, and as her mouth was dry from exposure, she closed it, and swallowed the hook two or three inches below the uvula. So soon as she discovered her situation, the whole family was assembled by her calls and cries of distress, *except little Charley*, who had dropped his pole in a panic, and, in provincial phrase, *mizzled.*

Some gentle efforts were essayed to remove the hook, both by the patient and some of the family; but being apprehensive of fixing the barb in the throat, they ceased all efforts, and despatched a messenger for Dr. E. Leroy Antony, who resided in the neighbourhood. When he arrived, and found that the hook was not fastened into the flesh, his fertile brain suggested a plan by which it could be removed safely, easily, and *without an operation.*

His plan was, to cut off the line within a foot or two of the mouth of the patient; then to drill a hole through a rifle bullet and drop it over the line, down on the hook. In order to fix the bullet on the point of the hook and maintain it firmly in that position, a reed was procured, the joints punched out, and then passed down over the line, and pressed firmly over the bullet. In this manner the hook, bullet, and reed, were all withdrawn at once, very easily, without any injury to the œsophagus or fauces.—*N. O. Medical Journal.*

